# Exploring Women's Experiences of Work Participation During Breast Cancer Treatment: A Qualitative Study

**DOI:** 10.1111/hex.70929

**Published:** 2026-10-22

**Authors:** Lucia Cadorin, Anna Fortin, Sonja Cedrone, Jessica Longhini, Matteo Danielis

**Affiliations:** ^1^ Direction of Health Professions, Centro di Riferimento Oncologico di Aviano (CRO) IRCCS Aviano Italy; ^2^ U.O.C. Oncologia, Azienda ULSS 6 Euganea Padova Italy; ^3^ Laboratory of Studies and Evidence Based Nursing, Department of Cardiac Thoracic, Vascular Sciences and Public Health, University of Padova Padova Italy

**Keywords:** breast cancer, cancer treatment, descriptive phenomenology, work experience, work participation

## Abstract

**Background:**

Breast cancer and its treatments significantly affect women's daily functioning, including their ability to engage in work. While return to work has been widely studied as an outcome, less is known about how work participation is experienced and negotiated during active treatment, particularly in relation to patients’ functioning and support needs.

**Aim:**

The aim of this study was to explore how women with breast cancer experience and make sense of work participation while undergoing treatment.

**Methods:**

A descriptive phenomenological study was conducted with 19 women receiving treatment for breast cancer at three hospitals in northeastern Italy. Data were collected through semi‐structured interviews and analysed using Colaizzi's seven‐step method.

**Results:**

Two main themes emerged: (1) ‘The individual‐focused nature of work experience,’ comprising three subthemes: personality traits shaping work perspectives, personal adaptation processes, and work as a source of emotional normality and identity continuity. (2) ‘Receiving support from the external context,’ including compassionate support acknowledging vulnerability and organisational adjustments facilitating work participation.

**Conclusions:**

Work participation represents a clinically relevant dimension of breast cancer care, reflecting the interplay between symptom burden, functional capacity, and psychosocial well‐being. Integrating work‐related considerations into routine clinical practice may support more comprehensive and responsive care during active treatment.

**Patient or Public Contribution:**

No patients or members of the public were involved in the design or conduct of the study. Participants contributed through individual interviews, and three subsequently took part in member checking of the preliminary thematic structure to enhance the credibility of the findings.

## 1 Introduction

Breast cancer remains one of the most significant global health challenges, with an estimated 2.4 million new cases and 694,000 deaths worldwide in 2024, and ranks as the most commonly diagnosed cancer among women in 164 countries [1]. Mortality rates have shown a consistent downward trend, with the most pronounced reductions recorded over the past decade, averaging annual decreases of 2%–3% in Denmark, Switzerland, the Netherlands, Norway, Sweden, Australia, the United Kingdom, and New Zealand [2]. Although the median age at breast cancer diagnosis in the U.S. is 64 years (https://seer.cancer.gov/statfacts/html/breast.html), meaning that half of those diagnosed are younger, a substantial proportion of women are still of working age. Therefore, the impact of the disease and its treatment on employment has become an increasingly important issue. In Europe, approximately 557,900 new breast cancer cases were reported in 2022, even if substantial differences exist between countries. While the disease predominantly affects middle‐aged and older women, a significant proportion of cases are diagnosed before typical retirement age [3]. Consequently, many women with breast cancer need to maintain their employment, either partly or fully, during treatment and/or return‐to‐work (RTW) following treatment. Although treatment‐related side effects can adversely affect well‐being and create barriers to employment, work remains a key aspect of quality of life for cancer patients and survivors alike, providing psychological, social, and financial benefits and helping women regain a sense of normality [4].

RTW was defined as having returned to paid work for any number of hours, either in the cancer survivor's original job or in alternative employment, within 1–24 months [5]. Work participation is a broad concept encompassing different aspects and stages of engagement in paid work, including having employment, maintaining productivity at work and returning to employment over time [6]. A substantial body of literature has examined survivors’ experiences of RTW, reinforcing its importance in survivorship care [5, 7, 8]. Work offered significant benefits, including a sense of identity and accomplishment, as well as a sense of normalcy, while also providing daily structure and social connection. At the same time, persistent physical limitations and functional symptoms adversely affected work performance, while RTW was generally perceived as easier and less stigmatising when clear workplace policies and employer support were in place. However, the meaning and value attributed to work may also change following a breast cancer diagnosis. Prospective evidence indicates that women may reorder their priorities following diagnosis, placing greater emphasis on their private life, with this shift influenced by clinical, psychosocial, and work‐related factors [9]. The systematic review by Tan and Shorey (2021) focused on the experiences of women with breast cancer while working or returning to work, thereby more fully encompassing the broader concept of work participation [4]. Women expressed a clear need for flexible treatment and work schedules to manage competing commitments more effectively, highlighting the importance of fostering a supportive and accommodating working environment in which flexibility and understanding are integral to sustainable work participation. More recently, Gershfeld‐Litvin et al. (2025) used a qualitative design to explore the experiences of 10 Israeli women who were working or returning to work while coping with cancer [10]. Participants described the need to negotiate treatment and rehabilitation alongside career demands, employer expectations, and work–life balance, suggesting that working during cancer involves an ongoing process of adaptation rather than a discrete RTW event.

Breast cancer treatment can be understood as a health–illness transition that disrupts women's bodily experiences, identities, social roles, and everyday routines [11]. Meleis’ Transitions Theory is relevant in this context, conceptualising transitions as processes of change through which individuals acquire new knowledge, modify their behaviours, and renegotiate their identities and roles within changing social settings [12]. Breast cancer‐specific studies describe this process as involving movement between health and illness, adaptation to changes in identity, and efforts to envision and negotiate a future beyond diagnosis and treatment [13]. From this perspective, work participation is not merely an employment outcome or a RTW date; rather, it is one domain in which the transition becomes visible, reflecting how women negotiate symptoms, life priorities, changing capabilities, professional identity, workplace expectations, and available support [9, 14].

Because this study explores women's lived experiences of working during active breast cancer treatment, work participation represents its primary conceptual focus, consistent with Meleis’ view of transition as an ongoing process rather than a single event. In the present study, work participation is used as an overarching term encompassing both maintaining employment during treatment and returning to work following a temporary interruption while treatment is still ongoing. Previous evidence has not always addressed this broader construct [4], has not always focused on breast‐cancer‐specific samples [10], and has not, to date, reported on the experiences of women in Italy. Addressing this gap is essential to developing effective, context‐specific interventions to support women's work participation during the active phase of care.

## 2 Methods

### 2.1 Study Design

This study adopts a descriptive phenomenological design to explore how women with breast cancer perceive and experience work participation in their everyday life. This approach focuses on capturing participants’ authentic lived experiences [15]. Two key processes are central to this method: first, bracketing, in which researchers identify and suspend their presuppositions (pre‐existing beliefs); and second, intuiting, through which they remain open to participants’ meanings [15]. The study was conducted in accordance with the Consolidated Criteria for Reporting Qualitative Research (COREQ) guidelines, available at https://www.equator-network.org/.

### 2.2 Objective and Research Questions

The study aimed to explore the experiences of women with breast cancer undergoing active treatment as they navigate work participation, including maintaining or returning to work during treatment. To this end, the study addressed the following research questions: ‘What are the experiences of women with breast cancer undergoing cancer treatment regarding work participation during active treatment?’ and ‘What forms of support do women perceive as helpful or lacking from healthcare professionals when attempting to maintain or return to work during active cancer treatment?’

### 2.3 Study Settings

This study was conducted in oncology day hospital settings in northeastern Italy. A day hospital is a care modality that allows patients to undergo diagnostic procedures and receive treatments through scheduled visits without overnight admission. Clinical activities, including chemotherapy, immunotherapy, radiotherapy, and supportive therapies, are delivered within a single day, enabling patients to return home after each session. The first setting included the oncology day hospital services of two hospitals within a healthcare trust in the Veneto region (Italy), serving a catchment area of approximately 936,000 inhabitants. The second setting was the oncology day hospital of a public, non‐profit cancer institute in the western Friuli region (Italy), which receives cancer patients from across Italy.

### 2.4 Sample and Procedure

Purposeful sampling was employed to enrol women with breast cancer of varying ages and diverse professional backgrounds (e.g., administrative assistant, engineer, manual worker) relevant to the phenomenon of interest [16].

The inclusion criteria required participants to be adults (≥ 18 years) with a diagnosis of breast cancer who were undergoing at least one form of cancer treatment (chemotherapy, pharmacological, surgical, or radiotherapy). Participants were required to be receiving care in an oncology day hospital setting, to have been in paid employment at the time of diagnosis, and to have maintained or returned to paid work during active treatment. They also had to be fluent in the researchers’ native language, and willing to share their experiences. Exclusion criteria included unemployment at diagnosis, permanent cessation of employment following diagnosis without returning to paid work during active treatment, psychiatric disorders, cognitive impairment, or terminal illness. Additional exclusion criteria were lack of fluency in Italian and voluntary withdrawal from the study [17, 18, 19]. There was no pre‐existing personal relationship between the research team and prospective participants at this stage.

### 2.5 Data Collection

Individual face‐to‐face interviews were conducted by members of the research team who were trained in qualitative methods between June 2024 and June 2025. The interviews were carried out by the first and second authors in a quiet and comfortable setting within the oncology wards of the participating hospitals. They followed a semi‐structured interview guide developed by the research team (Table 1). Examples of the questions include: (1) *Could you tell me about your work experience at this point in your life?* (2) *Could you talk about the factors that have made it easier for you to remain at work? Please provide examples*. (3) *How do you think work might influence your health condition?* The interview guide was pilot‐tested with the first two participants to assess the clarity and relevance of the questions. As no modifications to the interview guide were required following the pilot interviews, both interviews were included in the final sample. Each interview was audio‐recorded and subsequently transcribed verbatim. Interviews lasted between 31 and 58 min, with a mean duration of 40 min. Data saturation was monitored throughout data collection and analysis. After the 17th interview, the research team met to review the emerging findings and reached a consensus that no new significant statements, formulated meanings, or thematic insights had emerged from subsequent interviews. Two additional interviews were subsequently analysed using the same analytical procedure to assess the stability and completeness of the thematic structure. These interviews yielded variations within the existing thematic framework but did not generate new meanings or themes.

**Table 1:** Interview guide.

Question topic	Interview questions
Demographic and professional data	Collection of demographic and professional data
Work experience	Could you tell me about your work experience at this point in your life?
Work and health interaction	How do you think work might influence your health condition?
Facilitators of work continuation	Could you talk to me about the factors that have made it easier for you to remain at work? Please provide examples
Barriers to work continuation	Could you talk to me about the factors that have made it more difficult for you to remain at work? Please provide examples
Decision to continue working	How did you decide to continue working?
Workplace support	Could you tell me if you have been offered any support in the workplace? If so, could you provide some examples?
Healthcare professionals' role in support	How could the healthcare professionals who follow you help make your return‐to‐work experience easier and more positive?

### 2.6 Data Analysis and Rigour

Data analysis began with the verbatim transcription of each interview. To protect participants’ confidentiality, direct identifying information was removed and each participant was assigned a unique code (e.g., Patient 1 = P1). Age and occupation are reported alongside quotations to provide contextual information while preserving participant anonymity. Four researchers (L.C., A.F., J.L., M.D.) independently read the transcripts several times to gain familiarity with the data.

To support the descriptive phenomenological analysis, all researchers engaged in an ongoing bracketing process throughout data analysis. Before and during the analytical process, the research team held regular meetings to acknowledge and critically reflect on their preconceptions, clinical experiences, and expectations regarding work participation during breast cancer treatment, and on how these might influence data interpretation. Bracketing was therefore treated as an iterative and collaborative reflexive process that accompanied the development and refinement of the thematic structure. Each researcher initially identified significant statements independently across all transcripts. After every three to four interviews, the research team met to compare the identified significant statements, discuss their preliminary meanings, and resolve differences in interpretation through discussion until consensus was reached. This iterative process enabled the progressive refinement of the emerging thematic structure while maintaining consistency throughout the analysis. Once all interviews had been analysed, the research team conducted a final review of the complete thematic framework to ensure coherence, consistency, and agreement across the dataset.

The analytical process followed Colaizzi's (1978) [20] descriptive phenomenological method. Significant statements were identified, meanings were formulated through team discussion, and these meanings were clustered into thematic units, subthemes, and overarching themes. The preliminary thematic structure was first reviewed within the research team and subsequently through member checking with three participants who attended follow‐up appointments at the participating oncology services. Participants were presented with the preliminary themes and subthemes and invited to comment on whether they adequately reflected their experiences and whether any relevant aspects were missing or misrepresented. Participants confirmed that the findings adequately reflected their experiences, and no substantial modifications to the thematic structure were required following their feedback. Finally, an exhaustive description of the phenomenon was developed, from which its fundamental structure was formulated: work participation during breast cancer treatment emerged as a dynamic and personally meaningful process, continuously negotiated between individual meanings and adaptive capacities, on the one hand, and the support and constraints of the surrounding relational and organisational context, on the other. ATLAS.ti version 24 was used solely for data management and organisation, rather than for data analysis.

This study followed Lincoln and Guba's (1985) quality criteria to ensure rigour in qualitative research [16]. Credibility was supported through prolonged engagement with participants, iterative data analysis, investigator triangulation, regular analytical meetings, and member checking with three participants. Reflexivity was supported through transparent reporting of the research team's professional background and through ongoing reflexive discussion throughout the analysis. L.C. is a female PhD‐educated nurse with oncology and research experience; A.F. is a female Master of Nursing Science student and oncology nurse; J.L. is a female PhD‐educated assistant professor in nursing; and M.D. is a male assistant professor in nursing with a PhD. Transferability was addressed by providing a detailed description of the study context, participant characteristics, and sampling strategy. Dependability was ensured through an audit trail documenting methodological and analytical decisions throughout data collection and analysis, while confirmability was strengthened through researcher triangulation and documentation of the analytical process.

### 2.7 Ethical Considerations

Participants received written information about the study and provided informed consent through signed documentation. They were informed of their right to withdraw at any time. The data were anonymized and stored on a password‐protected personal computer, in accordance with the General Data Protection Regulation. Ethical approval was obtained from the Central‐East Veneto Territorial Ethics Committee (CET‐ACEV) on July 4, 2024 (study code: Rework; CET‐ACEV code: 6022/U6/24), and from the Regional Ethics Committee of the Friuli Venezia Giulia Region (CEUR‐FVG) on September 24, 2024 (study code: Rework CRO‐2024‐51; approval code: CEUR_FVG 0039716/P/GEN/ARCS).

## 3 Results

A total of 19 semi‐structured interviews were conducted. The sample comprised 19 women diagnosed with breast cancer. All participants had an active breast cancer diagnosis and were working at the time of interview. Time since first diagnosis ranged from one to 26 years; some participants had experienced a recurrence (e.g., one participant was first diagnosed in 1999 and later developed a recurrence in 2023, which led to her inclusion in the study). Participants described their cancer and work experiences in relation to their current, active diagnosis, not necessarily their original diagnosis. Regarding occupational physical activity, 12 participants (63.2%) were engaged in sedentary occupations, while 7 (36.8%) had physically active jobs. Full participant characteristics are presented in Table 2.

**Table 2:** Demographic characteristics of participants (*n* = 19).

PN	Age at interview (years)	Marital status	Children	Year(s) of cancer diagnosis	Therapy type	Work role	Occupational status	Work schedule (full‐time/Part‐time)	Employment sector	Occupational physical activity
1	50	Married	Yes	2014–2023	CT	Nurse	Employee	Full‐Time	Private	Physically active
2	48	Married	Yes	2023	CT	Administrative assistant	Employee	Full‐Time	Private	Sedentary
3	61	Married	Yes	2010–2023	CT	Engineering	Employee	Full‐Time	Private	Sedentary
4	55	Married	Yes	2007–2024	CT	Laboratory technician	Employee	Part‐Time	Public	Physically active
5	41	Married	Yes	2017	CT	Administrative assistant	Employee	Part‐Time	Private	Sedentary
6	59	Single	No	2022	CT	Administrative assistant	Employee	Full‐Time	Private	Sedentary
7	50	Single	No	2023	CT	Administrative and production manager	Employee	Full‐Time	Private	Sedentary
8	62	Married	No	1999–2023	CT	Nurse	Employee	Full‐Time	Private	Physically active
9	54	Married	Yes	2024	CT	Office management	Self‐Employee	Full‐Time	Public	Sedentary
10	55	Divorced	No	2023	CT, Immuno	Nurse	Employee		Public	Sedentary
11	60	Married	No	2023	SURG	Teacher	Employee	Full‐Time	Public	Sedentary
12	49	Single	No	2025	RT	Nurse	Employee	Full‐Time	Public	Sedentary
13	55	Married	Yes	2025	CT	Administrative assistant	Employee	Full‐Time	Private	Sedentary
14	59	Married	Yes	2025	RT	Manual worker	Employee	Full‐Time	Private	Physically active
15	54	Married	Yes	2025	CT	Administrative assistant	Employee	Full‐Time	Public	Sedentary
16	49	Married	Yes	2023	RT	Manual worker	Employee	Full‐Time	Private	Physically active
17	48	Partnered	No	2025	RT	Manual worker	Employee	Full‐Time	Private	Physically active
18	55	Married	Yes	2024	CT	Administrative assistant	Employee	Full‐Time	Mixed public–private	Sedentary
19	51	Partnered	Yes	2025	SURG	Nurse assistant	Employee	Part‐Time	Public	Physically active

*Note:* All participants had a primary diagnosis of breast cancer. Physical activity at work was classified according to the predominant work tasks reported by participants. Sedentary = predominantly desk‐based or seated work; Physically active = work mainly involving standing, walking, or manual tasks.

Abbreviations: CT, chemotherapy; Immuno, immunotherapy; PN, participant number; RT, radiotherapy; SURG, surgery.

The analysis identified two overarching themes describing patients’ experiences of work during cancer treatment: (1) ‘The individual‐focused nature of the work experience,**’** which explores personal traits, adaptation strategies, and the emotional significance of work; and (2) ‘Receiving support from the external context,’ which examines interpersonal support and organisational flexibility (Table 3).

**Table 3:** Statements of findings organised by themes and subthemes.

Themes	Subthemes	Statements of findings: work as…
The individual‐focused nature of the work experience	Personality traits that inform the individual's work perspective	Strong sense of duty and responsibility *‘[…] a sense of duty, of being present at work’ (P2: 48 years; administrative assistant)* *‘I never held back when it came to work, despite the illness, even though I feel tired at the end of my shift […]’ (P1: 50 years; nurse)* *‘I've always been in charge of organising my colleagues’ work, and that's a considerable responsibility’ (P14: 59 years; manual worker)* Long‐standing personal or family values *‘I never thought about taking long periods off work, perhaps because that's just my nature. I feel the need to give—it's something that really matters to me, it's a value’ (P16: 49 years; manual worker)* *‘I have always been very devoted to my work, deeply immersed in it, sometimes neglecting myself a bit’ (P7: 50 years; administrative and production manager)* Identity formation *‘I have my job, I still feel like a person’ (P9: 54 years; office management)* *‘Being a worker and contributing is an integral part of my identity’ (P15: 54 years; administrative assistant)* Meanings attributed to work during illness *‘I never thought about staying at home from work’ (P13: 55 years; administrative assistant)* *‘I never really considered the possibility of not returning to work’ (P11: 60 years; teacher)* *‘I'm someone who doesn't give up and just keeps going […] I'm very active, and I wanted to return as soon as possible […].* *I'm not someone who can stay still’ (P16: 49 years; manual worker)*
	Personal adaptation processes shape work tolerance	Adaptive capacity *‘I come to work, and if I feel well, I do what I can. I pace myself and my movements, because I still need to be careful’ (P17: 48 years; manual worker)* *‘I don't have physical difficulties that prevent me from working […]. I don't feel the need to stay at home’ (P6: 59 years; administrative assistant)* *‘I have a child, I have a mortgage, and somehow, I must make a living. If you don't receive 100% of your salary, it's not easy to get by. […] Besides necessity […] when you feel ready and think, ‘I'm not exactly at my best, but I can still manage to do something,’ you go [back to work]’ (P19: 51 years; nurse assistant)* Body listening *‘I come to work, I feel well, so I do what I'm able to do […] work is good for you. It's good psychologically and also in relationships, with the residents of the nursing home and with colleagues’ (P1: 50 years; nurse)* *‘I try to be one hundred percent autonomous. I try to stay as calm as possible, this is essential […] on the one hand I was fortunate because, physically, I was able to cope, and so I always went to work’ (P9: 54 years; office management)* Coping strategies activation *‘Work helps you not to think constantly about the illness […] it makes you feel like you're going back to normal’ (P1: 50 years; nurse)* *‘I tell the cancer: ‘I'm stronger than you.’ I fight’ (P8: 62 years; nurse)* *‘Having different human relationships helps me not to think, and eases the burden, let's say, of the weekly rhythm of my treatments’ (P15: 54 years; administrative assistant)* *‘The desire to feel healthy again, to feel like myself again—that is, normality’ (P17: 48 years; manual worker)* Personal challenge *At this stage of my life, I live day by day. I don't know what tomorrow will be like or what I will think […]. I didn't make a decision in advance. I just went on day by day, saw that I was managing, and then decided to keep working’ (P8: 62 years; nurse)* *‘The desire to feel healthy, to feel like myself again, to be strong’ (P17: 48 years; manual worker)* Internal resources mobilisation *‘I feel I can say, and this is my point of view, that something good always comes out of it. In some way, you grow anyway. It also depends a lot on one's character, on having a character that allows you not to see everything in a negative way. I believe I'm fortunate because this is the kind of character I have’ (P6: 59 years; administrative assistant)* *You become open to new things and possibilities’ (P12: 49 years; nurse)* Priorities recalibration *‘I don't put work at the centre of my life anymore’ (P7: 50 years; administrative and production manager)* *‘The work hasn't changed, but the way I approach it has. I now prioritize what is urgent and leave the rest for the following day’ (P10: 55 years; nurse)* *‘Through the illness, I realized what things are truly important and which ones are not’ (P10: 55 years; nurse)*
	Work is a subjective space of emotional normality and identity continuity	Emotional space *‘Going to work makes me feel normal, not sick’ (P15: 54 years; administrative assistant)* *‘When I'm at work, I feel like myself again […]’. ‘When I enter my workplace and take the reins of my job, I feel in charge. And that is a real remedy for me. Normality is a remedy’ (P8: 62 years; nurse)* Emotional relief *‘Having something demanding to focus on helps compared to staying at home’ (P10: 55 years; nurse)* *‘Being mentally occupied helps you not to dwell on negative thoughts’ (P19: 51 years; nurse assistant)* *‘Work is a psychological distraction […] being with colleagues instead of staying at home thinking about the worst’ (P3: 61 years; engineering)* *‘From a certain point of view, work distracts me; I can think about other things’ (P1: 50 years; nurse)* Psychological stability *‘Work is a kind of outlet […] it gives me the strength to go on, otherwise I think I would fall into depression’ (P4: 55 years; laboratory technician)* *‘If I had stayed at home every single day […]. I think I would have lost my mind. Working is better for me’ (P7: 50 years; administrative and production manager)* *‘Being focused on other commitments helped me detach my thoughts from treatment […] at the end of therapies I felt lost’ (P2: 48 years; administrative assistant)* Vulnerability *‘There are moments of tiredness during the day […] you have to learn how to manage it’ (P4: 55 years; laboratory technician)* *‘Working helps me say: I still have a semi‐normal life […] but illness forces you to rethink priorities’ (P5: 41 years; administrative assistant)* *‘I try not to think about it too much; otherwise, life goes on as before’ (P14: 59 years; manual worker)* *‘At the end of treatments, I felt lost […] and then what happens now?’ (P2: 48 years; administrative assistant)*
Receiving support from the external context	Compassionate support that acknowledges a person's vulnerability	Family support *‘I have a husband who supports me, so that definitely helps’ (P5: 41 years; administrative assistant)* *‘I ask my son to help me with small things, like unloading the dishwasher or making the bed […] he helps me, and so does my husband’ (P18: 55 years; administrative assistant)* *‘At home, I have the help of my family, my husband, and so on. At the same time, I've always been very independent’ (P9: 54 years; office management)* *‘My family is my greatest support’ (P19: 51 years; nurse assistant)* Healthcare partnership *‘I contacted the nurse to ask questions […]. I always found them available and kind in explaining things’ (P13: 55 years; administrative assistant)* *‘Everything was explained clearly, and the nurses were kind and helpful’ (P16: 49 years; manual worker)* *‘Doctors ask me if I need a few days of rest after therapy’ (P3: 61 years; engineering)*
	Organisational adjustments as a mediating process between the individual and the work environment	Flexible accommodation *‘If I'm tired, I sit down more instead of walking around the workplace’ (P7: 50 years; administrative and production manager)* *‘There are days when I feel nauseous, so I tell everyone I need to go out for a 5‐min walk, and no one says anything, not even my manager’ (P15: 54 years; administrative assistant)* *‘They told me to take my time, and that helps, because you know you can go to work without the obligation of having to feel well’ (P12: 49 years; nurse)* Autonomous task control *‘I organize myself, doing more demanding tasks in the morning and easier ones later’ (P17: 48 years; manual worker)* *‘They gave me the freedom to organize myself in the best way possible, both to manage my personal situation and to manage my work from home. In fact, I never received any remarks’ (P9: 54 years; office management)* *‘The main factor that helped me stay at work was the availability of the company and my autonomy in managing my tasks’ (P7: 50 years; administrative and production manager)* Part‐time arrangement *‘I'm fortunate because I have a part‐time contract and work only three days a week’ (P4: 55 years; laboratory technician)* *‘Chemotherapy on Wednesday, then two days at home to manage it’ (P12: 49 years; nurse)* Remote option *‘Working from home has given me a much calmer life’ (P5: 41 years; administrative assistant)* *‘I mainly work from home in smart working […] but I also like going in person, because contact with people matters’ (P18: 55 years; administrative assistant)* Supportive work community *‘They told me they missed me […] I felt welcomed’ (P15: 54 years; administrative assistant)* *‘I'm lucky to have a job that fulfils me and a very human, family‐like environment’ (P8: 62 years; nurse)* *‘My colleagues told me, ‘Do what you can, don't overdo it’. They protect me in that sense’ (P19: 51 years; nurse assistant)*

Abbreviation: P, patient.

### 3.1 The Individual – Focused Nature of the Work Experience

This theme captures how work during cancer treatment is primarily experienced through an individual lens, shaped by personal traits, values, emotional responses, and adaptive coping processes. Work is described not merely as an occupational activity but as a deeply subjective experience embedded in identity, motivation, and meaning‐making.

#### 3.1.1 Personality Traits That Inform the Individual's Work Perspective

Participants described how enduring personality traits, values, and beliefs strongly shape their perceptions of work and their approach to working during illness. Many reported a persistent drive to remain engaged in work, often describing increased commitment rather than withdrawal. For these participants, work is closely associated with a strong sense of duty and responsibility:

I don't approach my job without a sense of responsibility. I do it as I did before, trying to do it well.

(P6: 59 years; administrative assistant)

This dedication was frequently rooted in long‐standing personal or family values:

I don't even have the character to step back and think ‘I have the illness, so I do less’[…] I've never backed away from work, despite the illness. My father taught me that you have to work.

(P1: 50 years; nurse)

Several participants emphasised a persistent commitment to work as a defining aspect of their identity:

Work has always been an important point for me, because it defines you as a person.

(P4: 55 years; laboratory technician)

At the same time, participants described heterogeneous meanings attributed to work. While some continued to view work as a source of vitality and health, and as a way to divert attention from the illness and from concerns about an uncertain future:

For me, work is life, it is health. Especially during illness.

(P8: 62 years; nurse)

Others reported a re‐evaluation of priorities following the cancer experience, with work losing its centrality in favour of life, health, and personal time:

Now work has no value. I give value to my life… life has enormous value.

(P6: 59 years; administrative assistant)

For some participants, continuing to work was experienced as an almost unquestioned assumption, reflecting deeply ingrained personal attitudes toward work. As one participant explained:

I have always worked—from the time I received my diagnosis through chemotherapy. I was never on sick leave, except for the day of treatment.

(P13: 55 years; administrative assistant)

For another participant, the meaning attributed to work was also shaped by their position in life and career trajectory. Continuing to work was described as a way to maintain continuity and coherence with a long‐standing work history, particularly when approaching retirement.

I continue to work because I'm almost at retirement. That motivates me, because staying at home would make no sense. After everything I've been through, it doesn't make sense to stop working in these last months*.*

(P13: 55 years; administrative assistant)

#### 3.1.2 Personal Adaptation Processes Shape Work Tolerance

Participants described their ability to continue working during cancer treatment as the result of ongoing personal adaptation processes. Work capacity was not experienced as fixed but as something that required continuous monitoring and adjustment in relation to physical sensations, emotional states, and daily energy levels:

At work, I adjust my tasks according to how much energy I have.

(P17: 48 years; manual worker)

Several participants emphasised the importance of listening to their bodies and regulating work engagement accordingly:

I've always known I would go back to work… I listened to my body.

(P10: 55 years; nurse)

The presence or absence of treatment‐related symptoms played a key role in shaping perceived capacity to work. For some participants, the absence of significant physical symptoms enabled continued work participation:

I've always been well, so I've always continued to work.

(P13: 55 years; administrative assistant)

Conversely, some participants described maintaining or resuming work despite not feeling fully recovered, with financial necessity also influencing this decision.

Work was frequently described as an active coping strategy that supported psychological well‐being, providing distraction from illness‐related thoughts and helping maintain a sense of normality:

Working becomes a way of taking care of myself.

(P15: 54 years; administrative assistant)

For some participants, adaptation to work during illness was also experienced as a personal challenge that prompted reflection and change. The cancer experience was described as a turning point, leading to a more intentional and self‐directed approach to work and life priorities:

With the illness, I was faced with this challenge […] something that perhaps needed to be changed. I also saw it as, in quotation marks, a turning point to say that maybe I need to take control of my life*.*

(P5: 41 years; administrative assistant)

Several participants referred to internal resources such as resilience, determination, optimism, or faith as sustaining their ability to work during treatment:

You have to look ahead, hope, and have faith that things will turn out well.

(P19: 51 years; nurse assistant)

For others, adaptation also involved redefining priorities and setting new boundaries in relation to work demands:

Before, work was always the priority. Now the priority is myself.

(P10: 55 years; nurse)

#### 3.1.3 Work Is a Subjective Space of Emotional Normality and Identity Continuity

In contrast to the adaptive processes described above, participants emphasised the emotional and symbolic meaning that work assumed during cancer treatment. Beyond functional considerations, work was experienced as a subjective space of emotional normality, allowing participants to temporarily distance themselves from the illness and reconnect with a sense of continuity with their pre‐illness identity.

Several participants described work as an emotionally containing space that enabled them to feel normal, competent, and socially engaged, rather than primarily identified as patients:

When I'm at work, I don't feel ill. I feel healthy.

(P8: 62 years; nurse)

Work was also portrayed as providing emotional relief and psychological stability, helping participants regulate emotions and reduce illness‐related rumination:

Work helps you not to think about the illness… otherwise you stay home thinking about the worst.

(P7: 50 years; administrative and production manager)

For some participants, this sense of normality was closely linked to the preservation of personal and professional identity:

I have my job; I still feel like a worker.

(P9: 54 years; office management)

At the same time, participants acknowledged experiences of emotional vulnerability, fatigue, and moments of distress, which were particularly salient during transitional phases of the illness trajectory, such as the end of active treatment:

At the end of the therapies I felt lost… I cried for no reason… Sometimes you feel disoriented and more fragile, even when treatment is over.

(P2: 48 years; administrative assistant)

### 3.2 Receiving Support From the External Context

This theme describes how interpersonal relationship, healthcare, and organisational contexts provided, or limited, support that sustained participants’ ability to maintain or resume work during cancer treatment.

#### 3.2.1 Compassionate Support That Acknowledges a Person's Vulnerability

Participants highlighted the importance of compassionate, attentive, and human support from their social and healthcare networks during cancer treatment. Family support was frequently described as a primary source of emotional and practical support, providing reassurance and help in everyday life:

Family and friends… their closeness is really important.

(P12: 49 years; nurse)

Healthcare professionals (physicians and nurses) were perceived as supportive when they demonstrated availability, empathy, and individualised listening. Several participants described how feeling heard and treated with humanity contributed to emotional containment and reassurance during a particularly vulnerable period:

I found humanity on that side as well.

(P1: 50 years; nurse)

Practical support from healthcare professionals, particularly in relation to symptom management and treatment scheduling, was also described as indirectly facilitating work continuity:

When I explained the difficulties caused by certain symptoms, I was immediately given a solution, and that allowed me to continue working.

(P15: 54 years; administrative assistant)

At the same time, participants reported heterogeneous experiences regarding the perceived role of healthcare professionals in relation to work. While some described clinical support as enabling their ability to remain at work, others did not view healthcare professionals as directly involved in work‐related concerns. For these participants, clinical care and work were experienced as distinct domains:

I don't know whether a nurse can help me with work‐related issues. I see that they do their job well and give me all the necessary information.

(P11: 60 years; teacher)

#### 3.2.2 Organisational Adjustments as a Mediating Process between the Individual and the Work Environment

Workplace and organisational contexts emerged as key mediators between individual capacity and work participation during cancer treatment. Participants consistently described how organisational flexibility, accommodation, and supportive workplace cultures enabled them to sustain work despite physical and emotional challenges.

Flexibility in working hours, pacing, and rest opportunities was frequently identified as a crucial facilitating factor:

Flexibility in working hours, the possibility to rest if needed… that made everything easier.

(P2: 48 years; administrative assistant)

Several participants emphasised the importance of understanding supervisors and the ability to manage tasks and schedules independently according to daily energy levels:

They told me: ‘Your health comes first’… I was given complete freedom to manage my schedule.

(P9: 54 years; office management)

Organisational adjustments, such as part‐time work, task redistribution, and remote working arrangements, were described as essential for sustaining employment and reducing work‐related strain:

I work part‐time, and that's very important.

(P8: 62 years; nurse)

Participants also highlighted the role of colleagues and the broader workplace climate in mediating their work experience. Supportive, welcoming, and non‐judgmental environments contributed to a sense of belonging and emotional safety:

The environment was welcoming, and colleagues were supportive and open to dialogue.

(P11: 60 years; teacher)

In some cases, workplaces were described as ‘family‐like’ environments, where informal support and mutual understanding further facilitated continued work participation:

It's a very family‐like environment… everyone helps each other.

(P16: 49 years; manual worker)

Taken together, the themes and subthemes illustrate the interplay of individual and contextual factors shaping women's experiences of work participation during treatment. Figure 1 provides a visual synthesis of this thematic framework, highlighting the dynamic interaction between individual and contextual dimensions and the ongoing process of negotiation and adaptation over time.

**Figure 1:**
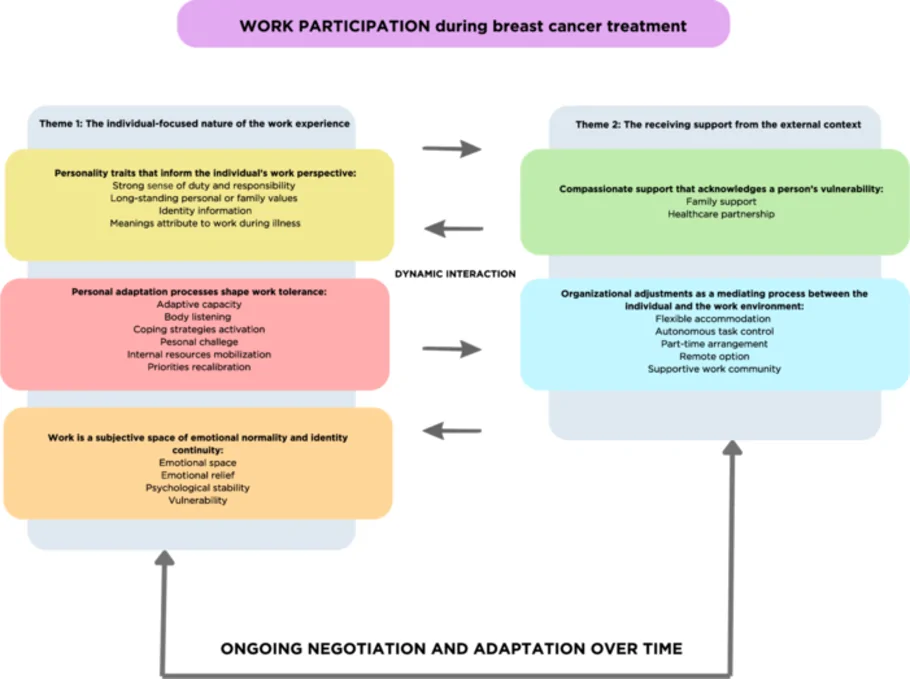
Thematic synthesis of work participation during breast cancer treatment.

## 4 Discussion

Breast cancer and its treatments are known to influence multiple domains of individuals’ lives, with well‐documented physical, cognitive, and psychological consequences that can compromise overall well‐being and quality of life [21]. Fatigue, nausea, and cognitive difficulties, including impairments in attention and memory, frequently interfere with daily functioning and work ability [22, 23].

Within this broader context of multidimensional life disruption, work represents a particularly sensitive domain. Existing literature has largely conceptualised work during and after cancer treatment in terms of symptom burden and return‐to‐work outcomes [19, 24, 25]. However, less attention has been paid to how individuals experience work while undergoing treatment and to the subjective meanings and personal resources that shape decisions to maintain or resume work activity.

The findings of the present study extend this body of research by foregrounding the lived experience of work participation during active cancer treatment. Rather than being understood solely as an outcome of clinical recovery or symptom resolution, work participation emerged as shaped by individual traits and values, personal adaptation processes, and emotional meanings related to normality and identity continuity. These individual dimensions were situated within relational and healthcare contexts, as well as workplace and organisational environments, which influenced opportunities and constraints for work participation during active treatment.

Building on this perspective, the findings highlight the central role of individual traits, values, and long‐standing attitudes toward work in shaping work experiences during cancer treatment. For many participants, work was closely linked to personal identity, self‐definition, and a sense of moral responsibility, rather than being experienced solely as an economic necessity. This resonates with previous research emphasising the role of work centrality and identity continuity in cancer survivorship, where maintaining work engagement can serve as a way to preserve a coherent sense of self in the face of illness‐related disruption [26, 27]. At the same time, meanings attributed to work were heterogeneous, ranging from continuity of work centrality to a redefinition of priorities following diagnosis. This variability aligns with qualitative research showing that cancer can both preserve and reshape work‐related values [28, 29].

Beyond individual values and identity‐related meanings, the findings of this study emphasise adaptation as a central process shaping patients' ability to tolerate and sustain work during cancer treatment. Participants described work tolerance not as a fixed capacity but as a dynamic and ongoing adaptation process that requires continuous self‐regulation in response to changing physical sensations, emotional states, and energy levels. This perspective aligns with previous research highlighting that cancer‐related symptoms, such as fatigue and cognitive difficulties, often fluctuate over time, requiring flexible and individualised approaches to work engagement [19, 25]. At the same time, adaptation remained closely tied to participants' desire to maintain a sense of continuity and normality, rather than disengage from work altogether. In this sense, work tolerance was shaped by a balance between acknowledging vulnerability and preserving agency, highlighting the nuanced and individualised nature of adaptation during cancer treatment [26, 30]. Consistent with previous studies, work was not experienced solely as a functional activity but as a meaningful space that supported emotional regulation, identity continuity, and a sense of normality during illness [26, 30]. Engaging in work allowed participants to temporarily distance themselves from the patient role and reconnect with aspects of their pre‐illness self, thereby counteracting the biographical disruption often associated with cancer. Work may thus serve as a stabilising element during cancer survivorship, contributing to psychological well‐being, self‐esteem, and perceived normality [29, 31]. At the same time, participants acknowledged moments of emotional vulnerability, fatigue, and distress, particularly during transitional phases such as the end of active treatment. These findings indicate that, while work can enhance quality of life and emotional stability, it may also coexist with ongoing psychological and emotional challenges related to the cancer trajectory [24, 28].

While individual traits, meanings, and adaptive processes played a central role in shaping work experiences during cancer treatment, the findings also underscore the importance of the external context in sustaining or constraining work participation. Participants' accounts illustrate how relational, healthcare, and organisational environments interact with personal resources, influencing both the feasibility and quality of continued work engagement during illness. Consistent with existing literature, relational support from family and close relationships was described as important in helping participants navigate the emotional and practical challenges of cancer treatment [32, 33]. Feeling emotionally supported and understood contributed to a sense of stability and reassurance, indirectly facilitating work participation. At the same time, participants highlighted the need to balance support with autonomy, as excessive concern or overprotection could be experienced as constraining, reflecting the ambivalent role of family support reported in chronic illness contexts.

Healthcare professionals were perceived as supportive primarily when they acknowledged emotional vulnerability and provided individualised, humane care. Being listened to and treated as a person rather than solely as a patient contributed to emotional containment during a particularly fragile phase of life, in line with previous research on person‐centred oncology care [34, 35]. However, the present findings also highlight in how the role of healthcare professionals is perceived in relation to work‐related issues. While some participants viewed clinical support as indirectly facilitating work through symptom management and treatment scheduling, others regarded healthcare and work as distinct domains, attributing limited relevance to healthcare professionals in relation to work participation. This heterogeneity points to a potential gap in the integration of clinical care and work‐related guidance within cancer care pathways.

Among external factors, the workplace and organisational context emerged as a particularly decisive mediator between individual capacity and actual work participation. In line with return‐to‐work literature, participants described organisational flexibility, accommodations, and supportive workplace cultures as key enabling conditions for sustaining work during cancer treatment [29, 36, 37]. Flexible schedules, task adjustments, part‐time arrangements, and remote work options allowed participants to align work demands with fluctuating physical and emotional resources, thereby reducing strain and anxiety related to performance expectations. Importantly, participants emphasised that informal practices, such as understanding supervisors, supportive colleagues, and autonomy in task management, were often as impactful as formal policies, echoing findings that relational workplace climates play a critical role in sustainable work participation [25]. Organisational adjustments were thus experienced not only as practical solutions but also as relational and symbolic signals of trust, recognition, and inclusion. These findings highlight the importance of integrating work‐related assessment into routine breast cancer care. Discussing work ability, treatment‐related symptoms, and potential adjustments during clinical encounters may support patients in balancing therapy and work demands. Such an approach may facilitate more individualised support for work participation during active treatment.

Our findings are consistent with Meleis' Transition Theory, which conceptualises health‐related transitions as dynamic processes involving changes in identity, roles, and relationships. Participants described work not simply as an occupational activity, but as an important means of maintaining a sense of identity, continuity, and psychological well‐being while adapting to breast cancer treatment. The influence of family, healthcare professionals, and workplace environments further reflects the role of individual and contextual factors in shaping the transition process.

### 4.1 Study Limitations and Strengths

This study has some limitations. Participants were recruited from three hospitals in northeastern Italy, operating within different healthcare contexts, which may have influenced care pathways and work experiences. The sample comprised women with breast cancer at different stages of the disease and treatment trajectory, which may have shaped how work was perceived and narrated. Furthermore, the sample was predominantly composed of married women, salaried employees, and participants engaged in sedentary occupations. Consequently, the findings may not fully reflect the experiences of women with different social or occupational circumstances, such as those living alone, engaged in physically demanding work, or self‐employed, who may face distinct challenges in balancing work participation with cancer treatment.

By design, this study focused specifically on women who maintained or returned to paid employment while undergoing active breast cancer treatment. Accordingly, the findings capture this particular lived experience and do not encompass the experiences of women who were unable to maintain or return to work during treatment. In addition, we did not systematically explore the extent to which maintaining or returning to work represented a voluntary choice or was driven by financial necessity. Consequently, economic strain and structural constraints that may have influenced participants' work participation may not have been fully captured in our analysis. Exploring the experiences of women who were unable to maintain or return to work, as well as the role of financial and structural factors in shaping work participation, could provide a complementary perspective on work and cancer during active treatment.

Member checking involved only three participants, as it was conducted during subsequent follow‐up appointments, which limited the opportunity to involve a larger number of participants. Nevertheless, credibility and confirmability were strengthened through investigator triangulation, collaborative data analysis, bracketing, and maintenance of an audit trail.

Furthermore, all interviews were conducted within the oncology setting, which may have influenced participants' accounts by making treatment‐ and healthcare‐related aspects more salient and potentially introducing social desirability bias, particularly when discussing coping strategies or support received from healthcare professionals. Although the interviews were conducted in a private and comfortable environment, future studies could offer participants a choice of interview setting to facilitate the expression of different dimensions of their work experience.

Despite these limitations, the descriptive qualitative design represents a key strength, allowing for an in‐depth exploration of participants' lived experiences, subjective meanings, and adaptive processes. Including participants across different treatment phases provided a dynamic understanding of work during cancer treatment, while the focus on personal narratives offered original, person‐centred insights into the work–cancer relationship.

Future qualitative research should explore work participation among more diverse occupational and social groups, including women engaged in physically demanding occupations and those who are self‐employed, as their experiences may differ substantially from those captured in the present study. Longitudinal studies following patients across different phases of treatment and survivorship could further clarify how the meaning attributed to work and supportive needs evolve over time. Finally, future research should evaluate interventions aimed at integrating work‐related support into routine oncology care and examine their impact on patient functioning and work participation.

### 4.2 Clinical Implications

The findings of this study highlight how women with breast cancer experience and attribute meaning to work participation during active treatment. Healthcare professionals should routinely explore work participation during clinical encounters, including patients' current work status, work‐related challenges, and concerns about balancing treatment and employment. These discussions may help anticipate treatment‐related difficulties and incorporate work‐related considerations into care planning. Given that participants attributed different meanings to work, healthcare professionals should also explore the importance that work holds for each individual, rather than assuming that it has the same relevance for all patients [9]. This person‐centred approach may help identify patients' priorities and guide individualised supportive care. When clinically appropriate, treatment planning and symptom management may also be aligned with patients' work‐related priorities, helping women balance treatment demands with meaningful work activities throughout the treatment trajectory.

Participants' accounts also underscore the importance of compassionate communication and attentive listening. Providing individualised information and acknowledging patients' vulnerability were perceived as central elements of supportive care.

The findings further emphasise the value of collaboration between healthcare services and workplaces. Flexible arrangements, such as task adjustments, part‐time work, or remote working options, were described as facilitators of continued work participation [38]. Clinicians may, where appropriate and with the patient's consent, facilitate communication between patients and employers by providing relevant information about treatment‐related functional limitations and the potential need for temporary workplace accommodations. When work‐related concerns extend beyond the scope of routine clinical care, referral to appropriate work‐related support services, where available, may also be considered.

Overall, work‐related support should be viewed as a shared process involving patients, healthcare professionals, and organisational contexts. From a transition perspective, integrating work‐related considerations into person‐centred cancer care may support women in adapting to changes in roles, identity, and everyday functioning throughout treatment.

### 4.3 Conclusion

This qualitative study documented the experiences of women with breast cancer in relation to work during active treatment, highlighting work participation as a complex and deeply personal experience shaped by individual traits, adaptive processes, personal meanings, and contextual conditions. Work participation was not determined solely by physical capacity but was closely tied to identity, a sense of normality, and the desire to maintain continuity with life before illness. At the same time, maintaining or returning to work required ongoing adaptation to fluctuating symptoms and emotional states, while relational, healthcare and organisational contexts played an important mediating role. Recognising this interplay between personal meanings, adaptive capacities, and contextual support may inform more person‐centred approaches to cancer care. Integrating work‐related considerations into routine oncology practice may help healthcare professionals recognise patients' individual priorities and support work participation through active treatment.

## Author Contributions

**Lucia Cadorin:** writing – original draft, writing – review and editing, validation, supervision, methodology, formal analysis, project administration, conceptualisation. **Anna Fortin:** writing – review and editing, writing – original draft, validation, project administration, methodology, investigation, formal analysis, conceptualisation. **Sonja Cedrone:** writing – review and editing, project administration, conceptualisation. **Jessica Longhini:** writing – original draft, writing – review and editing, validation, supervision, methodology, formal analysis, conceptualisation. **Matteo Danielis:** writing – original draft, writing – review and editing, validation, supervision, methodology, formal analysis, project administration, conceptualisation.

## Ethics Statement

This study was performed in line with the principles of the Declaration of Helsinki (2024). Approval was granted by the Central‐East Veneto Territorial Ethics Committee (approval codes: study code: Rework; CET‐ACEV code: 6022/U6/24), and from the Regional Ethics Committee of the Friuli Venezia Giulia region (study code: Rework CRO‐2024‐51; approval code CEUR_FVG 0039716/P/GEN/ARCS).

## Consent

Individual participant consent was obtained.

## Conflicts of Interest

The authors declare no conflicts of interest.

## Data Availability

The data supporting the findings of this study are accessible from the first author upon request.

## References

[1] H. Sung , A. M. Filho , M. Laversanne , et al., “Global Cancer Statistics 2024: Globocan Estimates of Incidence and Mortality Worldwide for 34 Cancers in 186 Countries,” CA: A Cancer Journal for Clinicians 76, no. 4 (2026): e70090, 10.3322/caac.70090. PMC13343830 42417444

[2] J. Kim , A. Harper , V. McCormack , et al., “Global Patterns and Trends in Breast Cancer Incidence and Mortality Across 185 Countries,” Nature Medicine 31, no. 4 (2025): 1154–1162, 10.1038/s41591-025-03502-3. 39994475

[3] X. Yu , C. Zhang , and M. Fu , “The European Burden of Breast Cancer in 2022,” European Journal of Surgical Oncology 52, no. 3 (2026): 111420, 10.1016/j.ejso.2026.111420. 41687168

[4] F. S. I. Tan and S. Shorey , “Experiences of Women With Breast Cancer While Working or Returning to Work: A Qualitative Systematic Review and Meta‐Synthesis,” Supportive Care in Cancer: Official Journal of the Multinational Association of Supportive Care in Cancer 30, no. 4 (2022): 2971–2982, 10.1007/s00520-021-06615-w. 34647131

[5] S. J. Tamminga , A. de Wind , M. A. Greidanus , et al., “Prognostic Factors for Return to Work in Breast Cancer Survivors,” Cochrane Database of Systematic Reviews 5, no. 5 (2025): CD015124, 10.1002/14651858.CD015124.pub2. PMC12056893 40331515

[6] M. Ravinskaya , J. H. Verbeek , M. W. Langendam , et al., “A General Framework for Selecting Work Participation Outcomes in Intervention Studies Among Persons With Health Problems: A Concept Paper,” BMC Public Health 22, no. 1 (2022): 2189, 10.1186/s12889-022-14564-0. PMC9701431 36435773

[7] P. Butow , R. Laidsaar‐Powell , S. Konings , C. Y. S. Lim , and B. Koczwara , “Return to Work After a Cancer Diagnosis: A Meta‐Review of Reviews and a Meta‐Synthesis of Recent Qualitative Studies,” Journal of Cancer Survivorship 14, no. 2 (2020): 114–134, 10.1007/s11764-019-00828-z. 31858379

[8] R. A. Cocchiara , I. Sciarra , V. D'egidio , et al., “Returning to Work After Breast Cancer: A Systematic Review of Reviews,” Work (Reading, Mass.) 61, no. 3 (2018): 463–476, 10.3233/WOR-182810. 30400123

[9] E. Caumette , A. Di Meglio , I. Vaz‐Luis , et al., “Change in the Value of Work After Breast Cancer: Evidence From a Prospective Cohort,” Journal of Cancer Survivorship 17, no. 3 (2023): 694–705, 10.1007/s11764-022-01197-w. 35267143

[10] A. Gershfeld‐Litvin , O. Vishnia , and T. Hanalis‐Miller , “Recovering or Working: Women's Experiences of Working While Coping With Cancer: A Qualitative Study,” Supportive care in cancer: official journal of the Multinational Association of Supportive Care in Cancer 33, no. 4 (2025): 289, 10.1007/s00520-025-09349-1. PMC11913926 40095125

[11] S. Keesing , L. Rosenwax , and B. McNamara , “The Implications of Women's Activity Limitations and Role Disruptions During Breast Cancer Survivorship,” Women's Health (London, England) 14 (2018): 1745505718756381, 10.1177/1745505718756381. PMC5808959 29409399

[12] A. I. Meleis , Transitions Theory Middle‐Range and Situation‐Specific Theories in Nursing Research and Practice (Springer Publishing Company, 2010).

[13] L. McCann , N. Illingworth , Y. Wengström , G. Hubbard , and N. Kearney , “Transitional Experiences of Women With Breast Cancer Within the First Year Following Diagnosis,” Journal of Clinical Nursing 19, no. 13/14 (2010): 1969–1976, 10.1111/j.1365-2702.2009.03134.x. 20936753

[14] G. Ruiz‐de‐Azua , I. Kousignian , I. Vaz‐Luis , et al., “Work Outcomes of Breast Cancer Survivors Who Returned to Work After Treatment: Canto Cohort,” supplement, European Journal of Public Health 32, no. S3 (2022): ckac131.211, Published 2022 Oct 25, 10.1093/eurpub/ckac131.211.

[15] S. Shorey and E. D. Ng , “Examining Characteristics of Descriptive Phenomenological Nursing Studies: A Scoping Review,” Journal of Advanced Nursing 78, no. 7 (2022): 1968–1979, 10.1111/jan.15244. PMC9320962 35405036

[16] Y. S. Lincon and G. E. Guba , Naturalistic Inquiry (SAGE Publications, 1985).

[17] I. Gruß , G. Hanson , C. Bradley , et al., “Colorectal Cancer Survivors' Challenges to Returning to Work: A Qualitative Study,” European Journal of Cancer Care 28, no. 4 (2019): e13044, 10.1111/ecc.13044. 31006931

[18] M. A. Greidanus , S. J. Tamminga , A. E. de Rijk , M. H. W. Frings‐Dresen , and A. G. E. M. de Boer , “What Employer Actions Are Considered Most Important for the Return to Work of Employees With Cancer? A Delphi Study Among Employees and Employers,” Journal of Occupational Rehabilitation 29, no. 2 (2019): 406–422, 10.1007/s10926-018-9800-z. PMC6531608 30027426

[19] W. Hou , Q. Li , X. Liu , Y. Zeng , and A. S. Cheng , “Exploring the Employment Readiness and Return to Work Status of Breast Cancer Patients and Related Factors,” International Journal of Nursing Sciences 8, no. 4 (2021): 426–431, 10.1016/j.ijnss.2021.09.001. PMC8488802 34631993

[20] P. Colaizzi , “Psychological Research and the Phenomenologist Views It.” in Existential‐Phenomenological Alternatives for Psychology, eds. In. R. Valle and M. King (Oxford University Press, 1978), 48–71).

[21] F. S. I. Tan and S. Shorey , “Experiences of Women With Breast Cancer While Working or Returning to Work: A Qualitative Systematic Review and Meta‐Synthesis,” Supportive Care in Cancer: Official Journal of the Multinational Association of Supportive Care in Cancer 30, no. 4 (2022): 2971–2982, 10.1007/s00520-021-06615-w. 34647131

[22] A. Lewandowska , G. Rudzki , T. Lewandowski , et al., “Quality of Life of Cancer Patients Treated With Chemotherapy,” International Journal of Environmental Research and Public Health 17, no. 19 (2020): 6938, 10.3390/ijerph17196938. PMC7579212 32977386

[23] N. G. Xunlin , Y. Lau , and P. Klainin‐Yobas , “The Effectiveness of Mindfulness‐Based Interventions Among Cancer Patients and Survivors: A Systematic Review and Meta‐Analysis,” Supportive Care in Cancer: Official Journal of the Multinational Association of Supportive Care in Cancer 28, no. 4 (2020): 1563–1578, 10.1007/s00520-019-05219-9. 31834518

[24] M. E. Schmidt , S. Scherer , J. Wiskemann , and K. Steindorf , “Return to Work After Breast Cancer: The Role of Treatment‐Related Side Effects and Potential Impact on Quality of Life,” European Journal of Cancer Care 28 (2019): 13051, 10.1111/ecc.13051. 31033073

[25] C. Maheu , M. Parkinson , C. Wong , F. Yashmin , and C. Longpré , “Self‐Employed Canadians' Experiences With Cancer and Work: A Qualitative Study,” Current Oncology 30, no. 5 (2023): 4586–4602, 10.3390/curroncol30050347. PMC10217428 37232805

[26] A. G. de Boer , S. J. Tamminga , J. S. Boschman , and J. L. Hoving , “Non‐Medical Interventions to Enhance Return to Work for People With Cancer,” Cochrane Database of Systematic Reviews 3, no. 3 (2024): CD007569, 10.1002/14651858.CD007569.pub4. PMC10913845 38441440

[27] X. Su , H. Sang , B. Kuai , P. Xue , and Y. Guo , “Experiences of Cancer Survivors Returning to Work Decision‐Making: A Meta‐Synthesis of Qualitative Studies,” Journal of Cancer Survivorship 19, no. 3 (2025): 1043–1058, 10.1007/s11764-023-01530-x. 38198115

[28] J. Xu , Y. Zhou , J. Li , et al., “Cancer Patients' Return‐To‐Work Adaptation Experience and Coping Resources: A Grounded Theory Study,” BMC Nursing 22, no. 1 (2023): 66, 10.1186/s12912-023-01219-7. PMC9999662 36899341

[29] W. Wu , H. D. Yackel , A. Salner , et al., “Work‐Health Balance of Cancer Survivors Returning to Work: A Meta‐Ethnography,” European Journal of Oncology Nursing 68 (2024): 102482, 10.1016/j.ejon.2023.102482. 38070450

[30] D. Von Ah and A. Crouch , “Relationship of Perceived Everyday Cognitive Function and Work Engagement in Breast Cancer Survivors,” Supportive Care in Cancer: Official Journal of the Multinational Association of Supportive Care in Cancer 29, no. 8 (2021): 4303–4309, 10.1007/s00520-020-05950-8. 33415361

[31] J. Yu , J. Smith , R. Marwah , and O. Edkins , “Return to Work in Patients With Head and Neck Cancer: Systematic Review and Meta‐Analysis,” Head & Neck 44, no. 12 (2022): 2904–2924, 10.1002/hed.27197. PMC9826518 36121026

[32] Y. J. Guo , J. Tang , J. M. Li , L. L. Zhu , and J. S. Xu , “Exploration of Interventions to Enhance Return‐To‐Work for Cancer Patients: A Scoping Review,” Clinical Rehabilitation 35, no. 12 (2021): 1674–1693, 10.1177/02692155211021706. 34227435

[33] I. Gabriel , D. Creedy , and E. Coyne , “Quality of Life and Associated Factors Among Adults Living With Cancer and Their Family Caregivers,” Nursing & Health Sciences 23, no. 2 (2021): 419–429, 10.1111/nhs.12823. 33605071

[34] G. R. Park and J. Kim , “Depressive Symptoms Among Cancer Patients: Variation by Gender, Cancer Type, and Social Engagement,” Research in Nursing & Health 44, no. 5 (2021): 811–821, 10.1002/nur.22168. 34254692

[35] S. de Oliveira Faria , G. Hurwitz , J. Kim , et al., “Identifying Patient‐Reported Outcome Measures (Proms) for Routine Surveillance of Physical and Emotional Symptoms in Head and Neck Cancer Populations: A Systematic Review,” Journal of Clinical Medicine 10, no. 8 (2021): 4162, 10.3390/jcm10184162. PMC8470145 34575271

[36] F. Mi and I. MI Nicoll , “Returning to Work After Cancer: Survivors’, Caregivers’, and Employers' Perspectives,” Psycho‐Oncology 28, no. 4 (2019): 792–798, 10.1002/pon.5021. 30720242

[37] D. Yagil , H. Goldblatt , and M. Cohen , “Dyadic Resources in the Return to Work of Cancer Survivors: Exploring Supervisor‐Employee Perspectives,” Disability and Rehabilitation 41, no. 18 (2019): 2151–2158, 10.1080/09638288.2018.1459885. 29631449

[38] W. Sun , K. Chen , A. Terhaar , et al., “Work‐Related Barriers, Facilitators, and Strategies of Breast Cancer Survivors Working During Curative Treatment,” Work (Reading, Mass.) 55, no. 4 (2016): 783–795, 10.3233/WOR-162449. PMC5571641 28059814

